# The UP150: A Multifactorial Environmental Intervention to Promote Employee Physical and Mental Well-Being

**DOI:** 10.3390/ijerph19031175

**Published:** 2022-01-21

**Authors:** Pietro Luigi Invernizzi, Gabriele Signorini, Raffaele Scurati, Giovanni Michielon, Stefano Benedini, Andrea Bosio, Walter Staiano

**Affiliations:** 1 Department of Biomedical Sciences for Health, University of Milan, 20129 Milan, Italy; pietro.invernizzi1@unimi.it (P.L.I.); gabriele.signorini@unimi.it (G.S.); raffaele.scurati@unimi.it (R.S.); giovanni.michielon@unimi.it (G.M.); Stefano.benedini@unimi.it (S.B.); 2 Polispecialistic Clinique San Carlo Srl., 20037 Paderno Dugnano, Italy; 3 Human Performance Laboratory, Mapei Sport Research Centre, 21057 Olgiate Olona, Italy; andrea.bosio@mapeisport.it; 4 Department of Physical Education and Sport, University of Valencia, 46010 Valencia, Spain

**Keywords:** motor efficiency, workplace, physical activity, self-determination, effort perception

## Abstract

Physical activity (PA) is a major health factor and studies suggest workplaces could promote PA by modifying office design, motivational strategies and technology. The present study aims to evaluate the efficiency of UP150, a multifactorial workplace intervention for the improvement and maintenance of the level of physical fitness (PF) and wellbeing. Forty-five employees were randomly divided into the experimental (EG) and control (CG) groups. The PF was assessed pre-post intervention using the *cubo fitness test* (CFT), the amount of PA was evaluated using the IPAQ questionnaire and accelerometers while the workload was assessed using the NASA-TLX questionnaire and psycho-physical health by using the SF-12 questionnaire. The EG worked in UP150 offices while the CG worked in their usual offices for 8 weeks. The EG and CG came back 4 weeks after the intervention for CFT retention. The EG improved CFT motor efficiency and the amount of moderate PA, while it reduced mental load. The EG retained reached motor efficiency levels 4 weeks after the intervention. No differences were found in IPAQ. The UP150 demonstrated to be a proactive environment and to be efficient in the promotion of PA, improving PF and mental health while decreasing mental load.

## 1. Introduction

The workplace represents one of the main causes of sedentarism and stress, which negatively affect the quality of life [1]. The incoming of the SARS-CoV-2 pandemic has changed usual working habits and has introduced the concept of smart working, which has caused a reduction in the costs for the companies accompanied by a reduction in employees’ working involvement and a decrease in working performances [2,3]. To act on the movement’s education and on employees’ motivation, a methodology is proposed that can determine an easy and non-traumatic transition from the classic workplace concept (based on constriction, stress and health risks due to a sedentary lifestyle) to a new workplace environment and office’s design concept, which consider the well-being and the caring of employees as central elements of companies’ welfare strategies [4].

The innovation of this methodology consists of the insertion of systems that can increase the motivation to perform physical activity, through the increase in autonomy, relatedness, and the positive perception of self-motor competence [5]. More specifically, a training system is proposed based on effort perception, which aims to integrate physical activity to professional working rhythms, promoting a conscious physical practice, adequate to the individual’s psycho-physical condition and to the working context.

The present research is an explorative study that aims to investigate the efficiency of a multi-component workplace intervention (UP150). In particular, the research aims to evaluate if the UP150 has efficacy in improving and maintaining the level of physical fitness, increasing the amount of weekly physical activity (reaching the WHO recommendations), decreasing the amount of perceived mental stress and decreasing the perceived mental load, using a training system based on self-perception and on the methodological principles of self-determination theory.

### Literature Review

The benefits of physical activity (PA) are widely known in the literature. Recent research reported that active people’s mortality risk is reduced by about 33% for all death causes, and about 35% for cardiovascular causes [6].

Furthermore, PA has an important role in the primary (healthy individuals), secondary (sick individuals) and tertiary (control of a diagnosed disease) prevention in most of non-communicable diseases (NCDs). Active lifestyles contribute in the prevention of coronary and atherosclerotic disease that are recognized as 31% of all death causes [7,8].

This health-generating effect has efficacy even in the prevention of metabolic syndromes, which has, as a consequence, the onset of diabetes and heart disease [9]. PA is known to be bound to the reduction in the risk of different cancer types and chronic obstructive pulmonary disease [10,11,12].

One of the main areas affected by PA is physical fitness, which is defined by Kilgore et al. [13] as the “possession of adequate levels of strength, endurance, and mobility to provide for successful participation in occupational effort, recreational pursuit, familial obligation, and that is consistent with a functional phenotypic expression of the human genotype”.

Based on the proposed definition, cardiorespiratory, muscular and flexibility are identified as the main components of physical fitness. More specifically, recent research assesses that cardiorespiratory training can reduce mortality by 11.6% for all death causes, 16% for cardiovascular disease and of 14% for cancer [14]. Muscular training is found to have positive effects on body composition, on insulin resistance, on self-esteem and on cardiovascular health [15].

Moreover, muscular training can improve and maintain the functions of musculoskeletal and neuromuscular systems, permitting an adequate autonomy through lifespan [16,17]. Flexibility training has positive effects on mechanical systems, increasing the articular ROM and muscles’ stretching capacity, preserving the independence of simple daily actions (such as washing, dressing and using the stairs) and reducing musculoskeletal issues that can produce articular pains (such as cervical pain or shoulder pain) and functional limitations that can be cause of falls (more frequents in the elderly) [18]. Furthermore, the research of Mueck-Weymann [19] reported that the heart rate variability of healthy adults can be positively affected by a stretching program of 7 weeks, probably through an activation of the parasympathetic nervous system. Another research concerning flexibility, conducted in the workplace, evidenced that an intervention of 10 min per day, for 5 days a week for 3 months, can reduce employees’ anxiety, pain, fatigue and, at the same time, increase mental health and vitality [20].

Moreover, the effects of physical activity on mental health have been well documented in recent years. It has been supposed that the antioxidant effect of physical exercise can positively impact mood disturbances related to oxidant stress [21]. Furthermore, PA effects promote the increase in some cerebral functions, such as learning and memory; in addition it can also reduce cerebral disease (such as Alzheimer or Parkinson) and age-related cognitive declines [22,23]. The effects of PA on mental health must be considered not only as a preventive tool, but even as an everyday instrument to improve the quality of life, thanks to physical exercise’s ability of giving a better body’s image, of reducing anxiety, stress and of promoting independence [24,25].

Based on previous affirmations, and on the existing literature, it can be asserted that physical inactivity can already represent by itself a risk factor for public health [26,27].

Considering the costs of this risk factor, the World Health Organization reported that the sanitary costs of each country attributable to physical inactivity range from 1% to 3% [28]. Recent data assess that the 27.5% of world population is not active enough, a percentage that increases to 36.8% when considering only higher-income countries [29].

The evidenced problems seem to have been worsened due to SARS-CoV-2 pandemic. An increasing amount of time spent in sedentary behaviors and a decrease in time spent in active or moderate activities has been highlighted [30]. Even the amount of steps performed during a typical week day has been affected by the restrictions imposed by country’s policies, evidencing a decrease of 27.3 % thirty days after WHO declared COVID-19 a pandemic disease [31]. One suggested solution is to promote physical activity and healthy lifestyles on the workplace, due to the elevated percentages of occupational diseases (70%) represented by NCDs caused by unhealthy behaviors [1].

It is important to consider that employees spend about half of their day in the workplace [32], and most common jobs require sedentary activities for a prolonged time, increasing the time spent in sedentary behaviors (10 ± 2 h/day during a week day).

From this point of view, it could be difficult for the employees to reach the recommended 150–300 min of moderate physical activities or the 75–150 min of intense physical activities [33].

The Global Action Plan on physical activity 2018–2030 [28], identifies the workplace as key to promoting physical activity by introducing active pauses, salutary physical activity and active urban mobility. Possible advantages caused by this type of intervention are the increase in employees’ wellness, productivity and soft skills (developing social relations and problem-solving capacities); the reduction in absenteeism; and cost reductions in public health [34,35].

In the same global action plan, it is highlighted that the workplace intervention must be focused on creating active environments, modifying the architectural structures of the workplace, and on creating active people, intervening on the employees’ lifestyle [28]. Based on the cited literature, the main research question is: *how to create active environments and active people?*

In a research conducted by Pronk in 2021 [36], four sociological intervention levels were identified:

- Individual level: based on individual consultancies, a dynamic work station (standing and sitting desks) and on the use of treadmill or stairs in active pauses or obliged routes in the office.
- Group level: introducing, in the working daily routine, group active pauses of 10 min, promoting online physical activities, walking groups and social support network.
- Environmental communication level: using printed billboards or personalized online messages (in order to promote healthy behaviors and physical activity), pedometers and informative campaigns.
- Policy and physical environment levels: promoting the creation of safe bicycle parking, the insert of changing rooms in the workplace and health programs.

In addition, the literature underlines that another important aspect to consider in the promotion of physical activity and creation of an active environment is motivation [37]. The lack of adequate motivation in conducting an active lifestyle could cause the early abandonment of the structured promotion program [38]. In order to prevent this issue, it is important to refer to a behavioral and motivational theoretical framework. In recent years, the self-determination theory (SDT) has been considered by many authors. This theory affirms that people are moved by three inner psychological needs: autonomy, competence and relatedness [39]. SDT has been widely suggested in the working context to develop the autonomy and proactivity of the employees in reaching working goals [40]. In addition, as evidenced by Deci and colleagues [41], autonomy, competence and relatedness can be considered as a mediator of health and wellness in the workplace and of physical activity [42]. The autonomy is the need to self-organize one’s own experience and one’s own behavior. Being autonomous means deciding and self-approving all actions in order to reach a specific goal [39]. In this psychological need, the goal setting theory [43] seems to be effective to promote motivation and autonomy.

In this theory, it is asserted that, when a task is perceived as too easy or too difficult, motivation decreases, while if the task is perceived as moderately difficult, motivation raises. This is supported by Brehm and colleagues who, moreover, assert that an increasing level of potential motivation due to a positive perception of the goal can increase the amount of effort dedicated in the achievement of the goal [44,45]. From this point of view, programs that aim to include physical activity promotion need to include adequate exercise in order to stimulate interest during practice. The competence is represented by the need of feeling efficient in one’s own social and physical world, the task required has to be adequate to the subject’s characteristics and the goals have to be clear. Relatedness is the need to feel connected with others, creating bonds and experimenting with belonging and intimacy; the individuals need to feel understood and positively evaluated.

Another important factor that may influence the employees’ motivation can be perceived in the offices’ design. It has been demonstrated that this latter factor has an important role in motivation enhancement [46]. In particular, the office’s design can reduce sitting time in employees acting on the environmental/spatial factor as workstations, furniture, office size, office density, shared spaces, corridors and stairs [47]. As previously explained, the increase in motivation is helped by the development of competence, which is composed by the acquisition of abilities and knowledge [48]. In order to sensibilize, educate and increase the knowledge of the employees about healthy lifestyles, recent researches have considered the inclusion of a new professional figure in the workplace environment, the wellness coach [49,50].

Wellness coaches have been shown to have great efficacy health risk prevention and in the promotion of active lifestyles even in workplace [51]. Moreover, the education to move and be active, given by the wellness coaches, is essential to create new abilities due to new routines and automatism, which could facilitate the approach to physical activity during working hours [52]. The inclusion of this new type of professional inside the working environment could contribute in enhancing the motivation to pursue physical activity and active lifestyles [51].

According to Commissaris and colleagues, a systematic review underlines that multi-component approaches (individual, organizational and environmental changes) can have more efficacy than single component approaches [53].

A study conducted by Maylor and colleagues [54] comprised a multi-component intervention named “Beat the Seat”, which aimed to reduce the amount of time spent in the sitting position during the workday, intervening on organizational, individual and environmental elements. The results showed that the intervention contributed to reduce the time spent in the sitting position and increased the amounts of transitions from the sitting to standing position and the number of steps performed during the workday.

Another multi-component intervention, proposed by Nooijen and colleagues [55], aimed to promote physical activity through the use of the company gym, walks during the lunch breaks and standing or walking meetings. The study underlines that, after 6 months from the start of the intervention, employees’ self-efficacy and motivation to approach physical activity increased significantly.

It is supposed that participants involved in the new proposed working environment could present higher levels of physical fitness and psychological well-being than a similar group in a standard working environment, considering both in-presence and smart working. Moreover, it is supposed that participants inserted in the new offices could increase the time spent in physical activities and maintain the improved physical fitness.

## 2. Materials and Methods

### 2.1. Sample

Forty-five employees volunteered to participate and were randomly divided into an experimental group (EG = 23) and control group (CG = 22). Only employees of the company that hosted the trial were included. Conversely, all the employees that presented psychological or physical disabilities were excluded from the experimental procedure. Moreover, the participants were excluded from the analysis if they did not complete all the expected evaluations or if they reached a percentage of work absenteeism greater than 10% during the experimental procedure.

During the experimental period, 5 participants (2 males and 1 female in the EG and 2 males in the CG) were no longer able to follow the procedures and were excluded from the analysis. At the end of the experimental procedure, both of groups were composed of 20 participants each (12 females and 8 males for each group). The participants of the EG were 31.7 ± 8.2 years old, with an average weight of 67.6 ± 17.0 kg and an average height of 1.7 ± 0.1 m (BMI= 22.6 ± 2.7 kg·m^−2^). Similarly, the participants of the CG were 32.0 ± 4.4 years old, with an average weight of 64.8 ± 9.9 kg and an average height of 1.7 ± 0.1 m (BMI= 22.9 ± 3.9 kg·m^−2^). The study was conducted in accordance with the declaration of Helsinki and was approved by the ethics committee of the University of Milan (14 September 2020, number 84/20).

### 2.2. Protocol

**Figure 1:**
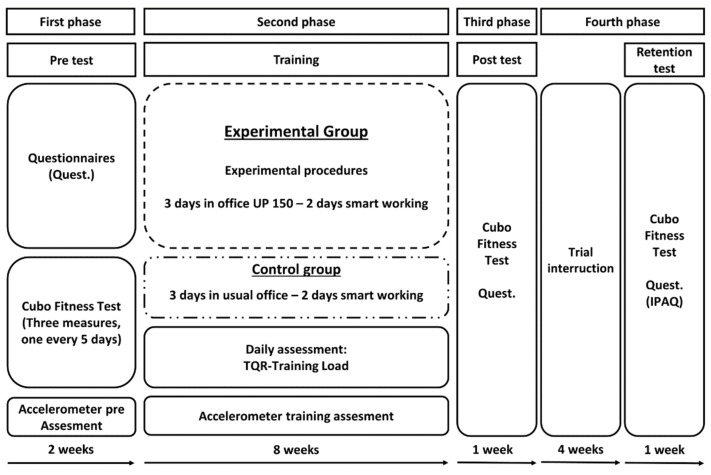
Protocol of the study.

The study was conducted following the structure of the randomized controlled trial. The first phase (pre-test) aimed to investigate the characteristics of the sample, proposing the *cubo fitness test* (CFT), and a set of questionnaires. The CFT was administered in 3 different sessions every 5 days to assess reliability. Moreover, in this phase, all participants wore the accelerometers for a week (detection 1). In the second phase (training), the participants were equally divided into two groups (EG and CG) with an equal amount of weekly physical activity measured in the first phase with the accelerometers and the international physical activity questionnaire. The EG worked three times a week in the new concept office and two times a week in smart working for eight weeks. During this period, the EG was subjected to the experimental procedures. The CG worked alternatively in their normal offices separately from EG (three times a week) and in smart working (two times a week) as usual. The CG was not allowed to interact with the procedures determined for the EG. In this phase, the physical activity of both groups was measured with the accelerometers, alternatively one week each, assessing three weeks for each group during the entire experimental period (detection 2, detection 3 and detection 4). In addition, all the participants had to report daily the total quality recovery value (at work entrance), the adapted Borg scale’s value (when leaving work) and the training load (calculated by multiplying the adapted Borg’s value by the working minutes of the day). After the expected eight weeks, in the third phase (post-test), the participants repeated the CFT and the set of questionnaires. In the fourth phase, all participants interrupted the experimental procedures for four weeks, and subsequently they repeated the CFT and the international physical activity questionnaire (retention test). The entire protocol is shown in Figure 1.

### 2.3. Assessment

All tests were administered in a dedicated test room inside the workplace office with a fixed temperature of 22 °C and with a standard humidity percentage of 40%. During the entire experimental period, participants followed the national indications for SARS-CoV-2 prevention.

### 2.4. Procedures

#### 2.4.1. Architectural Changes

The employees of the EG worked in new offices projected and developed by the specialized society “Progetto Design and Build S.r.l.”. The new concept of the office, named “Ufficio Proattivo 150” (UP150), was projected not only in order to include a set of physical activities stations that aimed to integrate movement during active pauses or during workflow, but even in order to create an environment that can increase the motivation of employees and social relationships. The following section explains the details of the physical stations.

Check in wall station: Wall fixed disposal, divided into three backlit segments (low, medium and high), which had to be touched while illuminated. The low segment had to be touched with both hands performing squats; the medium segment had to be touched while performing wall push-ups; and the high segment had to be touched rising on toes and reaching as high as possible. Each segment lit up for 10 s during which the employee had to perform the respective exercise. Each exercise was repeated two times, with a total duration of 60 s.

Steps station: The step station was structured as an informal meeting area, composed of steps, where employees could sit on. The structure presented two steps (of 50 cm and 90 cm) useful for performing different type of exercises (step-ups, push-ups at different heights; sit-squats and stretching for lower limbs).

Meeting rooms’ bike: The meeting rooms and the phone booth were equipped with a set of cycle ergometers that allowed to perform physical activity during meetings and work calls.

Break room’s treadmill: The break rooms were equipped with treadmills that allowed to walk and talk with colleagues during pauses.

Steppers (toilet, vending machines, standing desks): A set of steppers were placed in different office areas. The steppers allowed to log into many office workflow activities, such as washing and drying hands, buying coffee or snacks, or just stepping while working. To log into the activity, the employee had to perform at least 30 s of steps. The employee could also choose to bypass the procedure and to freely log into the activities without performing the exercise.

Rubber bands (break room and standing desks): A set of rubber bands with three different difficulties were placed in the break room and at the base of standing desks. These bands were useful to perform some simple exercises bound to muscular fitness for upper and lower limbs (at least 30 s for each exercise).

Reclining fitness bench: A dedicated area was equipped with a reclining bench that allowed the execution of stretching for lower limbs, sitting sit-ups with different inclinations of the bench and push-ups.

Wooden stick: The office’s common areas were equipped with wooden sticks. These sticks allowed to perform exercises for shoulder and upper limb mobility.

#### 2.4.2. App UP150

It is an application for mobility, developed by the society Business Integration Partners S.p.A. (Milan, Italy). The application includes the pocket trainer (PT), the training diary (TD), and physical activity score tools (PAS), which are explained in detail in the following sections. The App UP150 was developed to simplify the interaction process between the employee and the physical activity inside and outside the workplace. The application had to be activated at the entrance using the QR code linked to the “check in wall” or at home selecting a general check-in exercise. Before the “check in wall station” or any check in exercise, the application required the insertion of a value referred to the perception of total self-recovery using the TQR only once per day. Moreover, the App UP150 was able to connect the mobile phone to all the training stations using QR codes that permitted access to the description of the exercise, the suggested time and subsequently to the timer attached to the selected activity. The timer stopped when the employees decided to push the stop button on the mobile phone and the score reached in physical activity performed (see section on Physical Activity Score) was shown automatically. The same sequence could be used even outside the office, in this case the employee had to select a category of exercise (cardiorespiratory fitness, muscular fitness, articular fitness, sports activity and combined fitness) and to decide when to activate and to stop the timer. The TD system inserted in the application permitted to record all training activity, to compare the reached score with the weekly goal score assigned by the PT, and to give information about the duration and the perceived intensity of the exercise performed.

##### Pocket Trainer/Training Diary

The pocket trainer (PT) and the training diary (TD) are two components of a unique tool that aim to control and improve the index of motor efficiency (IME, see Section 2.5.1) and the participants’ lifestyle by increasing physical activity, consciousness, and perception of their own psycho-physical condition. The PT assigns a goal score, dependent to the IME that has to be reached (or overpassed) during the week, performing different types of physical activity. The goal score is normalized based on the level of the participants. The minimum target score is 150 points (level C), the intermediate target score is 225 points (level B), while the maximum goal score is 300 points (level A) according to WHO recommendations (from 150 to 300 min of moderate physical activity [33]).

##### Physical Activity Score

The physical activity score (PAS) is an instrument that allows codifying and scoring the physical activity. It was necessary to allow the participants to reach the weekly goal score and record the physical activity performed inside and outside the office. The score is determined by the duration of exercise (in minutes) multiplied by an effort perception’s coefficient. The coefficient is assigned as follows: the activities perceived from 0 to 3 (Light) on Borg’s scale adapted from NSCA (2012) [56] are considered as coefficient 1; the activities perceived 4 or 5 (moderate) are considered as coefficient 1.5; and all the activities perceived 6 or more (vigorous) are considered as coefficient 2.

#### 2.4.3. Wellness Coaches

The wellness coaches [49] were figures represented in this study by sport science graduate students. These specialists supported the employees during physical practice inside and outside the office. Furthermore, they personalized the exercises and the daily physical routine based on the employee’s necessities (previous disease, injury or specific goal or necessity, based on CFT results). Moreover, their role consisted of making the employees aware of the benefits of good practices and healthy lifestyles. The coaches were available online 7 days per week and in office 2 days per week to demonstrate and to explain the correct execution of the exercises.

#### 2.4.4. Self-Determination Methodology

The method used in this research followed the self-determination theory key points identified as the promotion of autonomy, competence, and relatedness [39]. Autonomy is promoted thanks to the possibility of choosing the type (cardiorespiratory, muscular, flexibility or combined physical fitness), the place (inside or outside the office) and the duration and the intensity of the physical activity to reach an assigned target score using the PT and the TD instruments.

Moreover, according to the considered literature, the architectural changes and the support of the application (App UP150) are thought to play an important role in autonomy promotion permitting to choose between many physical stations. Competence is guaranteed by the use of effort perception during physical activity. This method aimed to teach the employees how to practice physical fitness responsibly, respecting the participant’s internal load and avoiding the risk of an inadequate intensity effort. Relatedness is promoted by encouraging the interaction with the wellness coaches and with the other employees during the active pauses or during breaks. Moreover, relatedness is encouraged by the specifically designed new architectural environment elements as meeting room’s bike or break’s room treadmill. To prevent the possible interference of the social context on the physical activity motivation and to enhance the perception of being socially connected [57], wellness coaches were fundamental to help the transition from the classic office concept to the present approach. The wellness coaches were asked to create an adequate working climate where physical activity during working time is not considered an embarrassing moment, but, conversely, a well-accepted opportunity by all colleagues.

### 2.5. Measures

#### 2.5.1. Cubo Fitness Test

This test was administered to assess the physical levels of the employees in the office environment. The *Cubo Fitness Test* (CFT) [58] is composed by 5 submaximal tests based on effort and pain perception executed on a cube-shaped multifunctional instrument. These tests propose to evaluate cardiorespiratory fitness, muscular fitness and flexibility fitness, related to physical wellness and maintaining good health [59,60,61]. Each test gives a defined number of points depending on the test result. The maximal reachable points for each test was chosen taking into account the importance of each considered fitness category in preventing health and mortality risks. Cardiorespiratory fitness has been demonstrated to be fundamental in preventing cardiovascular diseases and other comorbidities, including hypertension, diabetes, heart failure and atrial fibrillation [62], representing the major cause of death worldwide [63,64]. A lower yet important contribution to health risk prevention is muscular fitness, which has been demonstrated to be efficient in reducing mortality risk [59,65]. Finally, flexibility fitness has been demonstrated to be effective in improving life quality [66], but no researches have demonstrated its efficacy in mortality risk prevention.

The index of motor efficiency (IME), resulting at the end of the five submaximal tests, ranges from 10 to 100 points. It summarizes the points reached in the 5 mentioned submaximal tests and was normalized based on age and sex. A score range lower than 33 points is considered a low level (level C), the score range included between 33 and 66 points is considered a medium level (level B) and the score range higher than 66 points is considered a high level (level A). The validity and the reliability of CFT were assessed in a previous research [58]. In the following section, all 5 submaximal tests are explained.

Ruffier test (RT) [67]: The participants had to sit and stand up from the cube with a frequency of 40 bpm for 30 times (or 45 s). During the test, we collected the resting hearth rate (HR0), the hearth rate (HR) immediately at the end of test (HR1) and the HR one minute after the end (HR2). The Ruffier index (RI) was calculated using the following formula: RI = (HR0 + HR1 + HR2 − 200)/100. Lower values of RI indicate a better performance. The height of the sitting position was modified using supports appropriately designed to maintain a 90° knee angle for each participant during the sitting activity. The effort perception was requested at the end of the test using the adapted Borg’s CR-10 scale [56]. The maximal acquirable score was 40 points.

Thirty second push-up test (PUT) [68,69]: The participants had to choose one of three difficulty levels in order to obtain an effort perception of “moderate” on the adapted Borg’s CR-10 scale. The levels were defined by the different distances from the ground where the hands’ support. Level 3 was the easiest (120 cm from the ground), followed by level 2 (60 cm from the ground) and level 1 (40 cm from the ground). After choosing, the test required the performance of the maximum possible number of push-ups in 30 s, with the subsequent assessment of target effort perception at the end of the test. The maximal acquirable score was 20 points.

Thirty second seated sit-up test (SUT) [68,69]: The participant had to choose one of three difficulty levels in order to obtain an effort perception of “moderate” on the adapted Borg’s CR-10 scale. The levels were defined by a different inclination of the seat’s back support. Similar to the PUT, Level 3 was the easiest (90° of inclination), followed by level 2 (45° of inclination) and level 1 (15° of inclination). The participant was requested to sit at the edge of the sitting area and to perform the maximum possible number of seated sit-ups in 30 s, with the subsequent assessment of target effort perception at the end of the test. The maximal acquirable score was 20 points.

Shoulder mobility test (SMT) [70]: The main tool of this test is a graduated stick. This instrument is marked with a precise measurement in centimeters starting from 0 in the middle point and increasing the measurement in equal increments in both directions. The participant had to hold the stick with both hands at the same distance from point 0. Starting with a large distance, participants had to perform a backward and subsequently a forward circle with upper limbs extended without losing grip. The participants were asked to repeat the test gradually reducing the hands distance, until they reached their limit without pain perception (value 100 of SIS scale [71]). The maximal acquirable score was 10 points.

Chair sit and reach test (SRT) [72]: The participant was asked to sit on the cube at the edge of the sitting area similarly to the SUT and to lay one leg on the graduated board while the other one was bent with an angle of 90° and with the foot on the ground. The centimeter placed on the graduated board was calibrated, making the point 0 to start next to the participant’s heel. Starting from this point, the centimeter presents two ranges of values: a positive one from heel to the ground and a negative one from heel to the participant’s hip. The participant was asked to slowly bend over (5 s) trying to reach or to overpass the heel with both hands performing the maximum stretching without pain (value 100 of SIS scale [71]), holding the position for 2 s while the evaluator measured the distance in centimeters from the hands’ middle fingers to the heel and subsequently to return in 5 s. The same measurement was performed for both of legs and the points were assigned using the mean value. The maximal acquirable score was 10 points.

#### 2.5.2. Questionnaires

##### International Physical Activity Questionnaire (IPAQ)

The IPAQ is a validated questionnaire that estimates the amount and the intensity of weekly physical activity performed by adults between 18 and 65 years old [73]. The questionnaire consists of 9 questions from which it was possible to obtain a score in Met referred to total activities. Moreover, thanks to the questionnaire, it was possible to estimate the weekly minutes elapsed in sedentary behavior (during working week and during weekend) or in light, moderate and vigorous physical activity. According to the literature, a total score lower than 700 Met was considered as inactive, a total score from 700 to 2519 Met was considered as active and a score up to 2519 Met was considered very active.

##### NASA Task Load Index (NASA-TLX)

The NASA-TLX is an evaluation instrument useful to investigate a perceived workload referred to a specific activity [74,75]. In the present research, it was requested to evaluate the workload referred to the previous working week [76]. The questionnaire permitted to evaluate 6 perceived loads: the mental demand (MD), the physical demand (PD), the temporal demand (TD), the effort (EF), the performance (PE) and the frustration (FR). Moreover, a total score (TS) summarized the overall workload.

##### Short Form Healthy Survey (SF-12)

The SF-12 is a validated questionnaire that aims to evaluate the psycho-physical health status of the participants [77]. It is a short version of SF-36 and consist of 12 questions. The SF-12 permits to estimate the self-reported health status by evaluating two indexes, identified as the physical component summary (PCS-12) and the mental component summary (MCS-12). The survey presents six questions that investigate the PCS-12 through physical activity, the limitations due to physical health, physical pain and general health, while six questions investigate the MCS-12 through social activities, vitality, emotional status and mental health.

#### 2.5.3. Accelerometers

In the present research, the triaxial accelerometers Axivity AX3 (Axivity Ltd., Newcastle upon Tyne, UK, 2013) were used in order to assess the amount of sedentary (lower than 1.5 Met), light (from 1.5 to 3 Met), moderate (from 3 to 6 Met) and vigorous (up to 6 Met) physical activities of the employees according to the literature [78]. The accelerometers’ measurements had a range of recorded acceleration of ±16 g and data were collected with a frequency of 100 Hz [79]. The accelerometers were worn on the wrist of the non-dominant hand [80] from Monday to Friday; the raw triaxial data were downloaded from the devices and exported using OmGUI software version 1.24 (Axivity Ltd., Newcastle upon Tyne, UK, 2013).

#### 2.5.4. Total Quality Recovery Scale (TQR)

In the present research, the TQR was proposed to evaluate the perceived recovery status of the employee before performing the CFT and during the entire working week. It is a validated scale that permits to evaluate the psycho-physical recovery referred to the last 24 h [81]. The TQR is composed by a range of value from 6 (very, very poor recovery) to 20 (very, very good recovery). The participant had to focus on his own recovery perception and, based on the scale’s verbal anchor, to give the most adequate value. The range of values from 12 to 14 (reasonable recovery) was considered adequate to perform the CFT.

#### 2.5.5. Training Load

Both the EG and CG were asked daily to record their effort perception, based on the adapted Borg’s scale, referring to the performed working hours. The training load was calculated by multiplying the effort perception reported by the working minutes performed [82].

### 2.6. Statistical Analysis

The normal distribution of data was conducted using the Kolmogorov–Smirnov test. The reliability of the CFT was assessed through the interclass correlation coefficient (ICC). The homogeneity of the analyzed groups was assessed by performing the unpaired *t*-test or the respective non-parametric Mann–Whitney *U* test. To verify the efficacy of the intervention, an ANOVA 3 × 2 (pre/post/retention × EG/CG) was performed for the CFT and IPAQ data, while an ANOVA 2 × 2 (pre/post × EG/CG) was performed for the NASA-TLX and SF-12 data. When a non-parametric analysis occurred, a Friedman test (to assess the intra-group differences) with a Mann–Whitney *U* test (to assess the inter-group differences) was chosen to replace the ANOVA 3 × 2, while the Wilcoxon test (to assess the intra-group differences) with the Mann–Whitney *U* test (to assess the inter-group differences) was chosen to replace the ANOVA 2 × 2. The delta values (pre-post) differences for each variable between the EG and CG groups were analyzed and compared using the unpaired *t*-test or the respective Mann–Whitney *U* test.

To analyze the accelerometer data, an ANOVA 4 × 2 (detection × EG/CG) was performed, while a Friedman test (to assess the intra-group differences) with a Mann–Whitney *U* test (to assess the inter-group differences) was chosen for the non-normally distributed data. During the three weeks of training of the experimental group, an ANOVA 3 × 2 (detection × condition) was used to assess differences in amounts of minutes of exercise during each of the three detections of training for the accelerometer and pocket trainer measurements of the intensity of the exercise. The independent sample *t*-test or respective Mann–Whitney *U* test were used to detect differences between the minutes and scores of physical activity performed inside and outside the office measured with the training diary. Significance was set at 0.05 (2-tailed) for all analyses.

To analyze the TQR and the training load data, an ANOVA 8 × 2 (evaluated weeks × EG/CG) was performed, while a Friedman test (to assess the intra-group differences) with a Mann–Whitney *U* test (to assess the inter-group differences) was chosen for the non-normally distributed data. The effect sizes for the repeated measurements using ANOVA were calculated as partial eta squared (η²p), using the small = 0.02, medium = 0.13 and large = 0.26 interpretation for effect size [83]. The effect size for the Mann–Whitney *U* test and Wilcoxon test was calculated as Pearson’s r, using the small = 0.1, medium = 0.3 and large = 0.5 interpretation of the effect size [84]. Moreover, the effect size for the Friedman analysis was calculated using Kendall’s W (W), using the small = 0.1, medium = 0.3 and large = 0.5 interpretation of the effect size [84]. All data analysis was conducted using the statistical packages for social sciences (SPSS version 21).

## 3. Results

### 3.1. CFT

All CFT tests results are shown in Table 1, while the CFT’s reliability data and the CFT’s RPE data are shown in Appendix A Table A1 and Table A2. CFT was found reliable for all its variables, and RPE measured in each test of CFT did not show any significative difference in between and within group analysis (*p* > 0.05).

#### 3.1.1. IME

**Figure 2:**
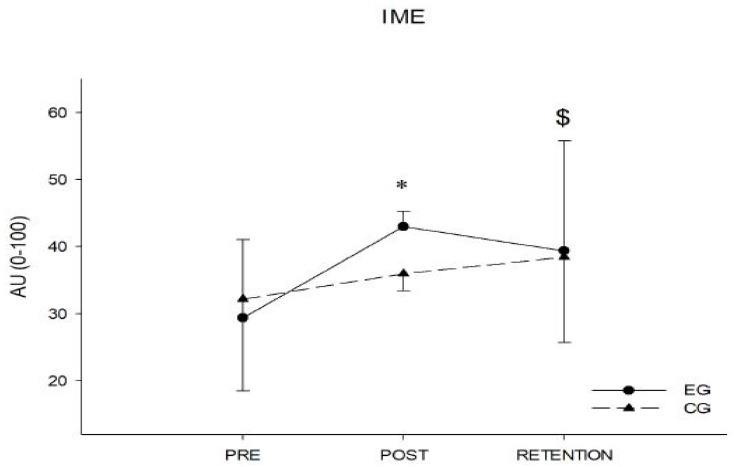
Index of motor efficiency analysis in the pre-post and retention phases. * = significant *p*-value (<0.05) in between groups analysis (EG vs. CG); $ = significant *p*-value (<0.05) in within EG analysis (pre vs. post vs. retention).

The index of motor efficiency test showed a significant interaction group by time (*p* = 0.008, η^2^ = 0.141), a significant main effect of time (*p* < 0.001, η^2^ = 0.351) and no significant main effect of group. Follow up tests revealed that, while the control group did not change over time from pre to post and from post to retention, the experimental group showed a significant increase in the test from pre to post (*p* < 0.001) and from pre to retention (*p* < 0.001), while no significant differences were found from post to retention, although there was a trend toward significance (*p* = 0.074). The analysis of the deltas showed a significant difference between groups regarding delta of the pre-post test (*p* < 0.001) and a trend to significance in the post–retention test (*p* = 0.052). Figure 2 shows the main results of IME.

#### 3.1.2. SMT

The shoulder mobility test showed a significant interaction group by time (*p* < 0.001, η^2^ = 0.243), a significant main effect of time (*p* < 0.001, η^2^ = 0.299) and no significant main effect of group. Follow up tests revealed that, while in the control group the shoulder mobility did not change over time from pre to post to retention, the experimental group showed a significant difference in the test from pre to post (*p* < 0.001) and from post to retention (*p* = 0.017). The analysis of the deltas showed a significant difference between groups regarding delta of the pre-post test (*p* < 0.001) and post–retention test (*p* = 0.034). All participants reported a value of SIS scale of 100, which corresponds to the maximum stretching without pain as requested.

#### 3.1.3. SRT

In the chair sit and reach test, a significant difference with the Friedman test (χ^2^(2) = 13.164, *p* < 0.001, *W* = 0.32) was found in the experimental group among the three different conditions (pre, post and retention). Follow up tests with Wilcoxon signed rank tests showed that there was a significant difference between pre and post conditions (*Z* = −3.024, *p* = 0.002, *r =* 0.67) and between post and retention condition (*Z* = −3.182, *p* = 0.001, *r =* 0.71). In the control group, no significant difference was found with the Friedman test. Mann–Whitney *U* test at post intervention showed no significant difference between groups.

The analysis of the deltas showed a significant difference between groups regarding the delta of the pre-post test (*Z* = −3.981, *p* < 0.00,1, *r =* 0.62) and a significant difference in the post–retention test (*Z* = −2.217, *p* < 0.027, *r =* 0.62). All participants reported a value of SIS scale of 100, which corresponds to the maximum stretching without pain as requested.

#### 3.1.4. SUT

In the sit-up test, a significant difference with the Friedman test (χ^2^(2) = 10.107, *p* = 0.006, *W* = 0.25) was found in the experimental group among the three different conditions (pre, post and retention). Follow up tests with Wilcoxon signed rank tests showed that there was a significant difference between pre and post conditions (*Z* = −3.268, *p* = 0.001, *r* = 0,75), while no significant difference was observed between post and retention conditions. In the control group, no significant difference was found with the Friedman test. Mann–Whitney *U* test at post intervention showed no significant difference between groups.

The analysis of the deltas showed a significant difference (*Z* = −2.427, *p* = 0.015, *r* = 0.38) between groups regarding the delta of the pre-post test. No significant difference was found for the post–retention test.

The RPE measured for the sit-up test at pre, post and retention did not show any significant differences over time and between groups.

#### 3.1.5. PUT

In the push up test, a significant difference with the Friedman test (χ^2^(2) = 11.414, *p* = 0.003, *W* = 0.33) was found in the experimental group among the three different conditions (pre, post and retention). Follow up tests with Wilcoxon signed rank tests showed a significant difference between pre and post conditions (*Z* = −3.361, *p* = 0.001, *r* = 0.75), while no significant difference was observed between post and retention conditions. In the control group, a significant difference was found with the Friedman test (χ^2^(2) = 17.492, *p* < 0.001, *W* = 0.51). Follow up tests with Wilcoxon signed rank tests showed a significant difference between pre and post conditions (*Z* = −3.420, *p* < 0.001, *r* = 0.76), while no significant difference was observed between post and retention conditions. Mann–Whitney *U* test at post intervention showed no significant difference between groups.

The analysis of the deltas showed no significant differences between groups regarding the delta of the pre-post test and post–retention test.

The RPE measured for the push up test at pre, post and retention did not show any significant interaction or main effects.

#### 3.1.6. RT

The Ruffier test did not show any significant interaction group by time or main effect of group. However, a main effect of time was detected (*p* < 0.001, η^2^ = 0.404). The Ruffier index decreased from pre to post (*p* < 0.001) with no differences between groups; however, it did not change between post and retention.

The analysis of the deltas showed no significant differences between groups regarding the delta of the pre-post test and post–retention test.

The RPE measured for the Ruffier test at pre, post and retention did not show any significant interaction or main effects.

### 3.2. Questionnaires

#### 3.2.1. IPAQ

No differences were found with the Friedman test in both groups for the physical activity questionnaire in any of the analyzed variables: total met, sedentary behavior during the weekend, sedentary behavior during the week, and light, moderate and vigorous physical activity. However, the Mann–Whitney *U* test revealed a significance for retention’s total Met values (*Z* = 1.986, *p* = 0.049, *r* = 0.31) and for post–retention delta’s light activities (*Z* = 2.222, *p* = 0.026, *r* = 0.35). All IPAQ data are shown in Table 2.

#### 3.2.2. NASA-TLX

No significant interactions or main effects were found for temporal demand, frustration and the total score that summarized the total workload.

The physical demand increased significantly (experimental: *Z* = −2.894, *p* = 0.004, *r* = 0.65; control: *Z* = −2.897, *p* = 0.004, *r* = 0.65) from pre to post uniformly with no differences in the groups. The Mann–Whitney *U* test revealed that there is no significant difference between groups at pre and at post. The delta analysis did not show any significant differences between the groups.

For the performance scale, Wilcoxon signed rank tests showed a significance difference between pre and post for the control group (*Z* = −3.508, *p* < 0.001, *r* = 0.78), while no differences were reported for the experimental group. The Mann–Whitney *U* test revealed a significant difference (*Z* = −2.192, *p* = 0.028, *r* = 0.34) between the groups at pre-test, while no significant difference between groups was detected at post-test. The delta analysis showed a significant difference in performance (*Z* = −2.882, *p* = 0.003, *r* = 0.46) between the two groups.

For the mental demand scale, Wilcoxon signed rank tests showed a significance difference between pre and post for the experimental group (*Z* = −2.913, *p* = 0.004, *r* = 0.65), while no differences were reported for the control group. The Mann–Whitney *U* test revealed a significant difference (*Z* = −2.041, *p* = 0.047, *r* = 0.32) between the groups at post-test, while no significant difference between groups was detected at pre-test. Delta analysis showed a trend toward significant difference (*Z* = −1.726, *p* = 0.087, *r* = 0.27) between the two groups.

**Figure 3:**
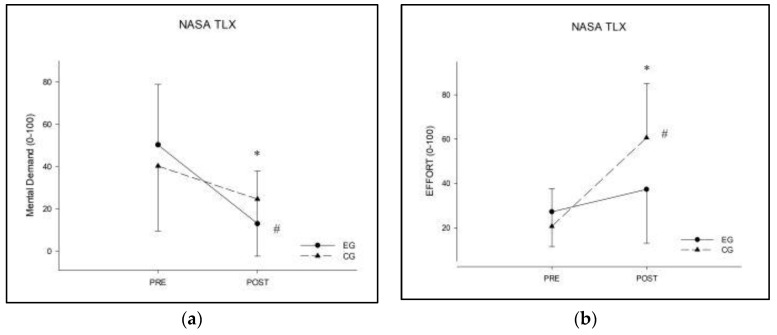
NASA-TLX analysis in the pre and post phases. (**a**) Analysis of effort; (**b**) analysis of mental demand. * = significant *p*-value (<0.05) in between groups analysis (EG vs. CG); # = significant *p*-value (<0.05) in within group analysis (pre vs. post).

For the effort scale, Wilcoxon signed rank tests showed a significance difference between pre and post for the control group (*Z* = −3.463, *p* < 0.001, *r* = 0.77), while no differences were reported for the experimental group. The Mann–Whitney *U* test revealed a significant difference (*Z* = −2.663, *p* = 0.007, *r* = 0.42) between the groups at post-test, while no significant difference between groups was detected at pre-test. The delta analysis showed a significant difference in effort (*Z* = −3.246, *p* = 0.001, *r* = 0.51) between the two groups. Figure 3 shows the effort and mental demand results. All NASA-TLX data are shown in Table 3.

#### 3.2.3. SF-12

The health questionnaire did not report any significant interaction (group by time) or main effects for the physical component (PCS). Regarding the mental component, a trend to interaction was found (*p* = 0.087, η^2^ = 0.070) and a significant main effect of time (*p* = 0.03, η^2^ = 0.154) was reported. The delta analysis showed a significant difference in the mental component (MCS) (*Z* = −2.102, *p* = 0.036, *r* = 0.33) between the two groups. SF-12 data are shown in Table 3.

### 3.3. Accelerometers

The accelerometers data are shown in Table 4.

No significant differences among weeks and for both groups were found for sedentary, light and vigorous minutes of exercises. No significant difference was found for the control group for moderate exercise among the four detections. A significant difference was found for moderate minutes of exercise in the experimental group among the four detections with the Friedman test (χ^2^(3) = 18.200, *p* < 0.001, *W* = 0.30). Follow up tests with Wilcoxon signed rank tests showed that the first detection was significantly different from detection 2 (*Z* = −2.215, *p* = 0.027, *r =* 0.50), detection 3 (*Z* = −2.605, *p* = 0.009, *r =* 0.58) and detection 4 (*Z* = −2.668, *p* = 0.008, *r =* 0.60), while detection 2 was significantly different from detection 3 (*Z* = −2.012, *p* = 0.044, *r =* 0.31) and detection 4 (*Z* = −1.961, *p* = 0.050, *r =* 0.31). No significant difference was found between detections 3 and 4. The Mann–Whitney *U* test did not show any significant inter group difference between the experimental and control groups for sedentary, light and vigorous minutes of exercise. However, in the moderate minutes category, a significant difference between the groups was found at detection 2 (*Z* = −2.336, *p* = 0.018). No other group differences were found in any other detection.

### 3.4. App UP150 (Experimental Group Procedures)

#### 3.4.1. Pocket Trainer vs. Accelerometer

No significant interaction or main effects were reported for the number of minutes during the three detections for the accelerometer and PT measurement of vigorous training. For light training, ANOVA revealed a significant main effect of condition (*p* = 0.046, η^2^ = 0.4090), showing that the experimental group reported in the PT more minutes in the light difficulty training (308.6 ± 201.9) than what the accelerometer index confirmed (187.5 ± 41.4). Contrary to that, in the moderate training, ANOVA detected a significant main effect of condition (*p* = 0.004, η^2^ = 0.674), showing that subjects in the experimental group reported less minutes in the moderate difficulty training (125.2 ± 110.2) compared to what the accelerometer index confirmed (427.7 ± 176.2). The results are reported in Table 5.

#### 3.4.2. Training Diary

From Table 6, it is possible to extrapolate that most of the exercises performed belonged to cardiorespiratory fitness (80% of the weekly activity), followed by muscular (9% of the weekly activity) and combined (9% of the weekly activity) fitness, while the less approached was the flexibility fitness (2% of the weekly activity). Nevertheless, flexibility fitness was practiced more inside the office than outside (59% vs. 41%), even without significant differences, while all other typologies of fitness were practiced more outside than inside the office (cardiorespiratory: 32% inside office, 68% outside; muscular: 29% inside office, 71% outside; combined: 25% inside office, 75% outside).

### 3.5. TQR

No significant interaction or main effects were reported for the total quality recovery scale. All TQR data are reported in Appendix A Table A3.

### 3.6. Training Load

No significant interaction or main effects were reported for the training load. All training load data are reported in Appendix A Table A3.

## 4. Discussion

### 4.1. Cubo Fitness Test

Based on the present data and comparing with the previous literature, CFT data were found reliable according to a previous research [58]. No differences were found in RPE values measured after each test of CFT, confirming the accuracy of the CFT instrument in performing measurements.

Analyzing the test results, CFT shows the efficacy of the intervention in the improvement of the level of IME. The EG passed from a low level (29.4 ± 13.7 a.u.) to a medium level (43.0 ± 15.6 a.u.). Consequently, it is reasonable to assert that the intervention has demonstrated efficacy in improving the EG’s general physical condition according to the multi-factorial intervention proposed by Maylor and colleagues [54].

As explained previously, the IME is the final CFT score, composed by the sum of the individual submaximal tests’ scores, and it is necessary to analyze the components that contributed to the overall improvement. The intervention significantly increased the levels of flexibility fitness (SMT and SRT) and muscular fitness (SUT). Considering flexibility fitness, the intervention improved both SMT and SRT in the EG, while the CG maintained the initial levels of flexibility. This effect could be due not only by the time spent performing flexibility exercises, but even by the concomitance of more factors, such as combined fitness (where a combination of muscular and flexibility fitness occurred) and muscular fitness [85]. In particular, muscular fitness has been demonstrated to have efficacy in improving flexibility when different muscle groups are involved alternatively [85]. Indeed, during the intervention, employees could freely approach different muscular exercises in the office and combined fitness permitted to increase the total amount of time spent performing activities related to flexibility fitness.

Analyzing the results of muscular fitness, the SUT showed an improvement in abdominal strength only for the EG. In the literature, it is well explained how the specific exercise of sit-up offers better results in the sit-up test than other exercises (curl-up or core stability exercises) [86,87]. From this point of view, the improvement in the SUT could be explained by the specificity of some exercises performed by the EG; indeed, sit-ups were one of the exercises proposed to the employees of the EG during the office intervention.

Differently, the results of PUT reported an increase in upper-limb muscular fitness for both groups. It should be noted that the intervention started soon before the end of the Italian lockdown restrictions for the SARS-CoV-2 pandemic (the intervention started 7 May 2021, while the main restrictions finished 24 May 2021). The reopening of gyms and sport centers could have permitted the improvement in some kind of fitness levels even in the control group. In particular, the push-ups could be defined as a commonly prescribed exercise that were recommended even to the computer worker population during the COVID-19 quarantine [88]. For this reason, PUT could have been influenced by a “background noise” that could have hidden the intervention efficacy. 

Even if both the EG and CG improved their cardiovascular fitness results, the intervention was not able to create differences between the two groups. The lack of differences could be explained by the duration of the intervention and the intensity of the performed exercises. A meta-analysis of Boulè and colleagues [89] considered eight weeks of cardiorespiratory training intervention as the minimum inclusion criteria. Nevertheless, the duration of the intervention program of the present research seems not enough to see more significant differences in the cardiorespiratory fitness of the EG employees. Even in this case, the lack of a significant difference between the two groups could be due, as described for PUT, to the end of the lockdown, which could have motivated even the CG to engage in more physical activity after a prolonged period of restrictions.

Nevertheless, data presented in the researches of Branch and colleagues, and Dunn and colleagues [90,91] showed that, at moderate intensity, marked results in cardiorespiratory fitness could be obtained after a wider period of training (12–24 weeks); considering the current pandemic situation, extending the period of intervention of the present study could bring more marked results.

Analyzing the retention effects, the employees of EG maintained the new acquired level of IME. Analyzing the results of the five submaximal fitness test, the EG retained the values of RT, PUT and SUT, while the values of SMT and SRT decreased. The results seem to agree with the research conducted by Chen and colleagues [92] that showed how different types of exercises could provide different effects after an intervention of 8 weeks and a successive rest of 4 weeks. In particular, the resistance and aerobic exercises seem to be efficient in maintaining positive effects after a period of interruption of the activities (the considered intervention included 2 days of training per week). The loss of flexibility could find explanation in the interruption of the stress-reduction effect promoted by the intervention [93].

Indeed, the results of our study demonstrated that the intervention reduced the perception of mental demand and improved the perceived mental health; all these factors could be considered as clues of the anti-stress effect of the intervention [93,94]. The absence of the intervention during the 4 weeks that followed the study could have contributed to increase the related muscular tone stress, as shown in a research based on visual display workers [95] and, consequently, could have reduced flexibility fitness.

### 4.2. Questionnaires

The proposed questionnaires highlight a positive effect of the UP150 on the mental psycho-physical components. Based on the significances found in the effort variable of the NASA-TLX, it can be concluded that the amount of practiced physical activities and, more in general, the intervention had a contribution to maintain the initial level of working effort perception conversely to CG, which increased the weekly working effort perception in the last period of training. This could be caused by the coping effect of the physical exercise during working hours [96,97]. This is supported by the research conducted by Wandel and Roos [96] who showed that physical activity could represent an effective coping strategy, especially for high position workers. This is confirmed by the lower level of working mental effort found in the EG in the last days of the experimental period. Moreover, the trend evidenced by the SF-12 seems to follow the line drawn by the NASA-TLX’s results; indeed, the MCS-12 showed a better improvement in the EG’s mental condition, confirming the positive effect of the experimental procedure.

The IPAQ did not show significant differences between the two groups in pre- and post-training conditions. The result seems to be contradictory with the higher number of minutes spent performing moderate physical activity recorded by the accelerometers in the EG. This lack of consistency between the accelerometers data and IPAQ datahas been previously seen by Dyrstad et al. [98]. More specifically, Dyrstad highlighted that, in the IPAQ questionnaire, the participants reported less sedentary time, less moderate intensity and a higher level of vigorous intensity physical activity than what was measured by the accelerometers.

Concerning the retention effect, despite an absence of significant intra-group differences in IPAQ, the results showed a difference in total Met values in favor of the control group, and the delta analysis seems to show that this difference could be due to a difference in walking activities [92]. During the experimental interruption, the participants did not receive any indication about the behavior to maintain concerning physical fitness and this might have caused the difference in outdoor activities [92]. It is necessary to mention that, in this period, the experimental group was not allowed to interact with any experimental procedure. Nevertheless, the employees of the EG were able to maintain the newly acquired levels of physical fitness during the retention test as evidenced in the previous paragraph.

### 4.3. Accelerometer

The analysis performed with the accelerometers underlines in EG a significant increase in minutes spent performing moderate physical activity and a decreasing trend of minutes spent in sedentary behavior. Even without significance, the EG evidenced a decrease of 5% of sedentary activities (from 84% to 79% of the total monitored time), while the CG did not show a similar trend (from 82% to 81%). Comparing the obtained data with the literature, it is possible to assess that the EG started with a mean time spent in sedentary behavior of 8.14 ± 0.8 h/day, considered as a range of increase in mortality risk (from 7.5 to 9.0 h/day) by WHO guidelines [33], and finished the experimental procedure with 7.8 ± 0.9 h/day, moving gradually away from the risk range.

### 4.4. TQR and Training Load

The lack of significances in TQR analysis seems to indicate that both of groups experienced similar recovery conditions, which corresponded to a “reasonable recovery”. The same can be asserted for the training load, which presented the same trend for both groups for the entire experimental period.

This last dataset appears to be at odds with the difference found in the effort values of the NASA-TLX, but it is necessary to specify that the NASA-TLX, as expressed by its protocol, is a self-reported questionnaire specifically that refers to the working task of the employee [74], and does not consider the physical fitness activity performed during working hours. Conversely, the training load was an evaluation of the general effort experienced during the working day which includes physical activity.

### 4.5. App UP150

The App UP150 permitted to collect data about the physical activity performed by the EG during the entire experimental period. In this way, it was possible to analyze and discuss the typology of fitness practiced, the reaching of the target score and the time spent conducting physical activity.

The app showed that all participants were able to reach the target score set by the CFT. Moreover, it is important to underline that about 75% of the target score (144.2 ± 74.4 a.u. of 191.9 ± 29.7 a.u.) was reached in the workplace during working hours. This could represent an important goal in this new workplace concept; physical activity was able to efficiently fit in the workflow, reaching the important percentage of recommended weekly physical activity [33]. The mean of the participants of the EG performed more than 350 min of physical activity during the week (summing the office physical activity and the outside office physical activity reported in the TD). Indeed, considering the accelerometer outcomes, it is possible to notice that the experimental group not only had an increased trend of moderate physical activity during the 8 weeks of intervention from 307.8 to 425.4 min (an increase of 3%), but even overpassed the minimum amounts of moderate minutes recommended of moderate physical activity. Nevertheless, it is possible to notice that the intensity of the activities perceived using the PT differs considerately from the intensity recorded by the accelerometers. The employees tended to underestimate the moderate physical activity, while tending to overestimate the light and vigorous physical activity.

Moreover, the results show that the participants of the EG interpretated most of the moderate intensity as light intensity. This phenomenon could be due to an easier perception of personal lower and upper bounds of effort, caused by the more shared meaning of ‘no effort’ and ‘maximal effort’ [99] during physical activity, while the different variations in effort could be more difficult to perceive. Another factor that could have contributed to increasing the difference between perceived and measured intensity of physical exercise is motivation. Based on Brehm’s motivational theory, if the task is perceived as adequate, the potential motivation that concerns the activity increases [44,45]. The increase in the employees’ motivation can influence the perception of practiced physical activity [44,45] and, as shown in the previous presented data, it is possible to notice that the minutes measured as moderate are more often perceived as light than vigorous.

### 4.6. Potential Limiations

The quarantine imposed to control the outbreak of SARS-CoV-2 could have influenced the participants’ lifestyle and consequently some results. Therefore, it is necessary to investigate the efficacy of the UP150 during a normal period, outside the restrictions imposed by the pandemic. Moreover, the small sample size did not permit to compare the effect of the intervention on females and males separately.

### 4.7. Practical Applications and Future Perspective

Due to the modification of the classic workplace concept, even caused by the current pandemic crisis, the office needs to change old schemes, adapting to new working modalities that include both in-presence and smart working [4]. The office intended as a simple physical space of work must change to follow the employees’ needs of organizational elasticity (both in-presence and smart working) and psycho-physical wellbeing [4]. From this new perspective, architectural changes could help the employees follow good practices for healthy behavior, facilitating the engagement with physical activity moments [100].

Moreover, the inclusion of a new professional figure in the form of the wellness coach could represent not only a facilitator of physical activity, but even a favoring element of social relationships that could have a positive impact on the working environment [101]. The technology must link the office (intended as physical space) with individual motivation (psychological level) and with opportunities of physical activities (fitness level) based on self-perception and regulation of effort. These three elements must be adaptable to the new working situation, switching from the in-presence to the smart-working modality and supporting the employees to develop healthy behaviors, even outside the working context.

In future investigations, a larger sample size could help to strengthen the effects of the experimental protocol.

Future research could focus the investigation on the longitudinal effects of the UP150 on the same proposed variables and could also prove the effectiveness of cardiorespiratory fitness and give better results in all other considered physical fitness categories [91]. Moreover, it could be useful to implement and verify the efficiency of new physical fitness stations linked to workflow moments or to active breaks, and the impact of the entire protocol on employees’ illness and stress related absenteeism [35].

## 5. Conclusions

The UP150 workplace intervention, based on architectural, technological, physical, and methodological components, seems to be efficient in the promotion of physical activity and an active lifestyle. In particular, the UP150 improved the employees’ index of motor efficiency, increasing flexibility fitness and part of muscular fitness. Considering the proposed questionnaires, the intervention decreased the work-based mental demand, maintained a fair level of working stress-related effort, evaluated with the NASA-TLX, and improved mental health, evaluated with the SF-12. Furthermore, the experimental procedure increased the number of moderate minutes of physical activity practiced during the working week, reaching and overpassing the minutes recommended by the literature.

## Figures and Tables

**Table 1:** Results of CFT test performed before the intervention (pre), at the end of the intervention (post) and 4 weeks after the end of the intervention (retention).

		Pre	Post	Retention	Delta 1	Delta 2
Ruffier (AU)	EG	13.1 ± 4.5	10.2 ± 16.2 #	9.9 ± 3.1	3.4 ± 2.2	0.3 ± 2.4
	CG	14.0 ± 5.2	11.2 ± 10.8 #	11.0 ± 3.6	2.8 ± 4	0.4 ± 3.6
30 s Push-up (AU)	EG	3.5 ± 1.9	6.6 ± 11.6 #	5.41 ± 3.1	3.1 ± 2.9	0.6 ± 3.1
	CG	3.2 ± 1.1	6.1 ± 10.0 #	5.7 ± 2.4	2.9 ± 2.5	0.4 ± 2.6
30 s Seated Sit-Up (AU)	EG	6.3 ± 3.2	9.4 ± 3.1 #	7.8 ± 4.2	3.3 ± 3.2 *	0.3 ± 2.6
	CG	7.0 ± 3.4	8.8 ± 4.0	9.6 ± 3.7	1.0 ± 2.4	1.0 ± 3.2
Shoulder mobility (cm)	EG	52.4 ± 9.8	44.0 ± 4.7 #	46.8 ± 9.3	7.6 ± 5.8 *	2.2 ± 4.7 *
	CG	49.3 ± 8.4	47.0 ± 3.0	47.5 ± 8.4	0.5 ± 3.7	1.2 ± 3.6
Chair sit and reach (cm)	EG	−5.0 ± 13.0	−0.8 ± 10.0 #	−2.5 ± 13.6	5.0 ± 7.2 *	4.6 ± 5.4 *
	CG	−0.4 ± 10.9	1.4 ± 11.6	−2.2 ± 12.2	1.2 ± 3.6	0.5 ± 3.7
Index of Motor Efficiency (AU)	EG	29.4 ± 13.7	43.0 ± 2.3 #	39.4 ± 16.4	14.4 ± 8.7 *	3.8 ± 8.4
	CG	32.2 ± 11.7	36.0 ± 2.6	37.4 ± 12.5	3.1 ± 10.5	3.0 ± 10.1

* = significant *p*-value (<0.05) in between groups analysis (EG vs. CG); # = significant *p*-value (<0.05) in within group analysis (pre vs. post vs. retention). Delta 1 is calculated with post–pre, while delta 2 with retention–post. Data are shown as mean ± standard deviation. Data of delta 1 and delta 2 are expressed in absolute values.

**Table 2:** Results of IPAQ performed before the intervention (pre), at the end of the intervention (post) and 4 weeks after the end of the intervention (retention).

			Pre	Post	Retention	Delta 1	Delta 2
IPAQ	Vigorous Activity (Met)	EG	822.9 ± 1182.3	802 ± 1047.1	1294.1 ± 1729.8	34.0 ± 871.7	501.2 ± 1290.7
		CG	830.5 ± 789.8	1302.2 ± 1060.5	1793.7 ± 1854.5	377.8 ± 1311.6	562.4 ± 2150.7
	Moderate activity (Met)	EG	701.0 ± 1105.8	859.2 ± 1320.6	828.2 ± 1512.7	147.2 ± 706.1	140.0 ± 1905.2
		CG	634.3 ± 795.2	377.8 ± 261.8	818.9 ± 966.1	238.9 ± 819.4	430.6 ± 1033.4
	Walking activity (Met)	EG	1604.6 ± 3145.3	1672.4 ± 3788.3	657.5 ± 720.4	40.0 ± 1235.5	1116.5 ± 3486.7 *
		CG	754.9 ± 720.4	815.4 ± 565.3	1211 ± 1086.7	12.8 ± 905.1	437.2 ± 1017.3
	Sedentary activity during working day (Met)	EG	506.8 ± 166.1	432.5 ± 188.0	411.8 ± 228.5	72.5 ± 154.8	15.9 ± 156.8
		CG	488.1 ± 150.3	450.0 ± 137.4	429.5 ± 186.6	42.8 ± 168.6	28.2 ± 120.6
	Sedentary activity during weekend (Met)	EG	184.3 ± 103.7	153.3 ± 122.0	144.7 ± 108.9	34.3 ± 119.1	6.2 ± 123.3
		CG	233.8 ± 188.1	216.7 ± 204.7	162.1 ± 105.3	29.4 ± 284.4	63.5 ± 188.2
	Total (Met)	EG	3128.5 ± 4781.4	3333.6 ± 5250.0	2779.8 ± 3332.9 *	221.2 ± 1785.4	755.3 ± 4721.0
		CG	2219.6 ± 1708.1	2495.4 ± 1301.2	3823.6 ± 2574.1	126.1 ± 1711.7	1430.1 ± 3023.0

EG = experimental group; CG = control group. * = significant *p*-value (<0.05) in between groups analysis (EG vs. CG). Data are shown as mean ± standard deviation. Delta 1 is calculated with post–pre, while delta 2 with retention–post. Data of delta are expressed in absolute values.

**Table 3:** Results of the NASA-TLX and SF-12 questionnaires performed before the intervention (pre) and at the end of the intervention (post).

			Pre	Post	Delta 1
NASA-TLX	Mental Demand	EG	50.3 ± 28.5	13.0 ± 24.9 # *	36.1 ± 44.5
		CG	40.3 ± 30.7	24.6 ± 27.0	15.9 ± 41.1
	Physical Demand	EG	8.6 ± 16.6	24.3 ± 21.5 #	15.3 ± 25.0
		CG	5.7 ± 9.0	19.1 ± 20.9 #	15.6 ± 18.8
	Temporal Demand	EG	44.7 ± 27.3	55.5 ± 20.0	11.2 ± 32.6
		CG	45.2 ± 25.5	49.2 ± 23.8	7.1 ± 29.1
	Performance	EG	22.0 ± 13.7 *	19.0 ± 21.1	2.8 ± 23.7 *
		CG	32.8 ± 18.3	9.7 ± 12.7 #	24.9 ± 19.1
	Effort	EG	27.2 ± 15.7	37.3 ± 24.4 *	9.0 ± 30.4 *
		CG	20.6 ± 17.1	60.6 ± 24.6 #	43.4 ± 29.9
	Frustration	EG	29.7 ± 35.2	34.8 ± 24.7	4.1 ± 35.5
		CG	27.2 ± 33.1	34.7 ± 23.5	11.2 ± 34.4
	Weighted sum	EG	12.2 ± 4.1	12.3 ± 2.8	15.3 ± 25
		CG	11.5 ± 4.3	13.2 ± 3.9	15.6 ± 18.8
SF-12	PCS12	EG	53.5 ± 7.2	55.3 ± 5.2	1.8 ± 6.3
		CG	56.1 ± 5.4	54.7 ± 5.7	1.4 ± 7.0
	MCS12	EG	37.5 ± 9.8	46.6 ± 8.2 #	9.1 ± 8.0 *
		CG	39.2 ± 9.0	42.3 ± 12.2 #	3.1 ± 13.5

* = significant *p*-value (<0.05) in between groups analysis (EG vs. CG); # = significant *p*-value (<0.05) in within group analysis (pre vs. post). EG = experimental group; CG = control group. Data are shown as mean ± standard deviation. Delta 1 is calculated with post–pre. Data are shown as mean ± standard deviation. Data of delta are expressed in absolute values.

**Table 4:** The outcomes of the accelerometers used in the intervention.

		CG	EG
Intensity		Mean ± SD	Mean ± SD
Sedentary minutes	Detection 1	2334.5 ± 321.4	2442.2 ± 252.6
	Detection 2	2353.5 ± 165.0	2309.2 ± 331.6
	Detection 3	2317.3 ± 294.7	2343.9 ± 262.4
	Detection 4	2382.3 ± 272.5	2332.4 ± 269.6
Light minutes	Detection 1	209.0 ± 55.0	177.2 ± 51.8
	Detection 2	200.3 ± 56.3	189.6 ± 55.7
	Detection 3	212.1 ± 55.8	202.1 ± 65.9
	Detection 4	221.8 ± 46.8	208 ± 51.5
Moderate minutes	Detection 1	300.9 ± 139.0	307.8 ± 176.7
	Detection 2	281.7 ± 171.6	355.4 ± 124.8 #*
	Detection 3	345.9 ± 200.6	392.9 ± 153.7 #§
	Detection 4	314.7 ± 117.3	425.4 ± 175.9 #§
Vigorous minutes	Detection 1	10.3 ± 20.3	6.1 ± 13.1
	Detection 2	6.6 ± 13.6	3.7 ± 5.0
	Detection 3	8.1 ± 12.1	4.5 ± 6.5
	Detection 4	7.3 ± 8.8	4.2 ± 5.5

EG = experimental groups; CG = control group. Detection 1 represents the pre-assessment; detection 2 represents the first measurement during the intervention; detection 3 represents the second measurement; detection 4 represents the third measurement during the intervention. * = significant *p*-value (<0.05) in between groups analysis (EG vs. CG); # = significant *p*-value (<0.05) in within group analysis (difference with detection 1); § = significant *p*-value (<0.05) in withing group analysis (difference with detection 2).

**Table 5:** The outcomes of the App UP1–0—Pocket Trainer used in the intervention.

	Accelerometer	PT	EG—Accelerometer	EG—PT
Intensity			Mean ± SD	Mean ± SD
Light minutes	Detection 2	Detection 1	189.6 ± 55.7	326.9 ± 248.1 *
	Detection 3	Detection 2	202.1 ± 65.9	342.1 ± 175.7 *
	Detection 4	Detection 3	208 ± 51.5	316.8 ± 199 *
Moderate minutes	Detection 2	Detection 1	355.4 ± 124.8	127.9 ± 139.8 *
	Detection 3	Detection 2	392.9 ± 153.7	118.8 ± 82.3 *
	Detection 4	Detection 3	425.4 ± 175.9	74.9 ± 80.9 *
Vigorous minutes	Detection 2	Detection 1	3.7 ± 5.0	15 ± 21.9
	Detection 3	Detection 2	4.5 ± 6.5	25 ± 50.4
	Detection 4	Detection 3	4.2 ± 5.5	27.4 ± 72.3

Data are shown as mean ± standard deviation. PT’s Detection 1 corresponds to the first measurement performed with the accelerometer during the second phase (detection 2); PT’s detection 2 represents the second measurement performed with the accelerometer (detection 3); PT’s detection 3 represents the third measurement (detection 4). The PT measurements shown represent the minutes of activity perceived as light, moderate and vigorous and reported in the App UP150 by the EG. * = significant *p*-value (<0.05) in between groups analysis (PT vs. accelerometer).

**Table 6:** App UP150—Training diary mean outcomes.

App UP150—Training Diary (Mean ± SD)			
	Total	Inside Office	Outside Office
Cardiorespiratory Fitness (Min)	314.9 ± 103.6	99.6 ± 59.0	214.8 ± 110.5 *
Muscular Fitness (Min)	35.1 ± 52.8	10.2 ± 7.8	25.0 ± 52.2
Flexibility Fitness (Min)	8.3 ± 8.5	4.9 ± 4.2	3.4 ± 5.4
Combined Fitness (Min)	35.2 ± 46.7	8.9 ± 7.0	26.3 ± 44.2
Total (Min)	394.8 ± 132.6	124.2 ± 65.6	269.9 ± 149.4 *
Cardiorespiratory Fitness (Points)	346.8 ± 103.7	112.3 ± 61.8	233.9 ± 115.4 *
Muscular Fitness (Points)	47.9 ± 70.2	13.0 ± 9.7	34.8 ± 70.3
Flexibility Fitness (Points)	9.2 ± 9.4	5.5 ± 4.8	3.7 ± 6.0
Combined Fitness (Points)	62.1 ± 73.4	12.7 ± 12.7	49.4 ± 68.8 *
Total score (Points)	467.3 ± 149.9	144.2 ± 74.4	322.3 ± 175.6 *
Target score (Points)	191.9 ± 29.7		

Data refer to the mean weekly number of minutes and points accumulated. The table shown data refer to physical activity performed inside the office (inside office), outside the office (outside office) and the total physical activity obtained summed the inside and the outside office activities. * = significant *p*-value (<0.05) in between groups analysis (inside office vs. outside office).

## Appendix A

**Table A1:** Reliability results of the CFT.

	Session 1	Session 2	Session 3	ICC
Ruffier (AU)	14.4 ± 4.9	13.6 ± 5.1	12.6 ± 4.8	0.950
Chair sit and reach (Cm)	−3.4 ± 10.5	−3.2 ± 12.3	−0.9 ± 15.0	0.944
Shoulder mobility (Cm)	52.4 ± 9.8	51.2 ± 9.5	47.3 ± 8.6	0.965
Thirty second Seated Sit-Up (AU)	5.8 ± 3.3	7.3 ± 4.0	7.3 ± 3.7	0.804
Thirty second Push-up (AU)	3.1 ± 1.7	3.1 ± 1.5	3.9 ± 1.9	0.804
Index of Motor Efficiency (AU)	27.3 ± 12	30.7 ± 13.4	34.8 ± 14.1	0.957

Sessions 1, 2 and 3 represent the three sessions performed previously to the intervention. Data are shown as mean ± standard deviation.

**Table A2:** Results of the CFT’s RPE analysis.

		Pre	Post	Retention
Ruffier (AU)	EG	5.3 ± 0.9	4.9 ± 1.2	4.9 ± 1.1
	CG	4.8 ± 0.7	4.8 ± 0.8	4.9 ± 0.9
Thirty second Seated Sit-Up (AU)	EG	4.2 ± 0.7	4.3 ± 0.6	4.1 ± 0.8
	CG	4.1 ± 0.6	4 ± 1	4.2 ± 0.7
Thirty second Push-up (AU)	EG	4.8 ± 0.6	4.8 ± 1.1	4.5 ± 0.8
	CG	4.7 ± 0.6	5.4 ± 0.9	4.8 ± 0.6

EG = experimental group; CG = control group. Data are shown as mean ± standard deviation.

**Table A3:** TQR and Training Load outcomes.

		CG	EG
		Mean ± SD	Mean ± SD
TQR	Pre	14.5 ± 1.7	13.8 ± 2.2
	Week 1	14.1 ± 1.4	13.7 ± 1.9
	Week 2	14.0 ± 1.6	14.2 ± 2.0
	Week 3	13.8 ± 2.0	14.7 ± 1.7
	Week 4	13.7 ± 1.4	13.8 ± 1.9
	Week 5	13.6 ± 1.7	14.2 ± 2.4
	Week 6	13.5 ± 2.2	14.2 ± 2.4
	Week 7	13.3 ± 1.7	14.2 ± 1.5
	Week 8	13.6 ± 1.5	14.1 ± 2.3
	Post	14.0 ± 2.3	13.5 ± 2.6
	Retention	14.7 ± 1.9	15.3 ± 2.7
Training Load	Week 1	2980.7 ± 793.5	3555.6 ± 1101.7
	Week 2	3210.2 ± 649.3	3313.9 ± 1184.3
	Week 3	2906.4 ± 838.1	3208.7 ± 815.8
	Week 4	3280.6 ± 660.2	3298.1 ± 1083.3
	Week 5	3189.4 ± 783.5	3284.2 ± 773.9
	Week 6	3155.3 ± 785.4	3469.3 ± 635.6
	Week 7	3002.3 ± 879.9	3316.7 ± 780.5
	Week 8	3337.4 ± 730.2	3375.1 ± 878.0

TQR values of pre, post and retention are referred to the measurements performed immediately before the CFT.

## References

[1] WHO (2010). Healthy Workplaces: A Model for Action.

[2] Bolisani E., Scarso E., Ipsen C., Kirchner K., Hansen J.P. (2020). Working from home during COVID-19 pandemic: Lessons learned and issues. Manag. Mark..

[3] Chanana N., Sangeeta (2020). Employee engagement practices during COVID-19 lockdown. J. Public Aff..

[4] Sica R. (2021). Dall’employee Experience All’employee Caring: Le Organizzazioni Nell’era Post COVID-19.

[5] Ryan R.M. (2012). The Oxford Handbook of Human Motivation.

[6] Nocon M., Hiemann T., Müller-Riemenschneider F., Thalau F., Roll S., Willich S.N. (2008). Association of physical activity with all-cause and cardiovascular mortality: A systematic review and meta-analysis. Eur. J. Prev. Cardiol..

[7] Alves A.J., Viana J.L., Cavalcante S.L., Oliveira N.L., Duarte J.A., Mota J., Oliveira J., Ribeiro F. (2016). Physical activity in primary and secondary prevention of cardiovascular disease: Overview updated. World J. Cardiol..

[8] Douglas Darden B.S., Caroline Richardson M.D., Elizabeth AJackson M. (2013). Physical activity and exercise for secondary prevention among patients with cardiovascular disease. Curr. Cardiovasc. Risk Rep..

[9] Saklayen M.G. (2018). The global epidemic of the metabolic syndrome. Curr. Hypertens. Rep..

[10] Warburton D.E.R., Bredin S.S.D. (2017). Health benefits of physical activity: A systematic review of current systematic reviews. Curr. Opin. Cardiol..

[11] Tong X., Chen X., Zhang S., Huang M., Shen X., Xu J., Zou J. (2019). Bone angiogenesis. Biomed. Res. Int..

[12] Oliveira J.S., Pinheiro M.B., Fairhall N., Walsh S., Franks T.C., Kwok W., Bauman A., Sherrington C. (2020). Evidence on physical activity and the prevention of frailty and sarcopenia among older people: A systematic review to inform the world health organization physical activity guidelines. J. Phys. Act. Health.

[13] Kilgore L., Rippetoe M. (2007). Redefining fitness for health and fitness professionals. J. Exerc. Physiol. Online.

[14] Imboden M.T., Harber M.P., Whaley M.H., Finch W.H., Bishop D.L., Kaminsky L.A. (2018). Cardiorespiratory fitness and mortality in healthy men and women. J. Am. Coll. Cardiol..

[15] Westcott W.L. (2012). Resistance training is medicine: Effects of strength training on health. Curr. Sports Med. Rep..

[16] Pereira F.D., Batista W.O., Furtado H.L., da Silva E.B., Júnior E.D.D.A. (2011). Functional autonomy of physically active and sedentary elderly women: Comparative causal study. Online Braz. J. Nurs..

[17] Marcos-Pardo P.J., Orquin-Castrillón F.J., Gea-García G.M., Menayo-Antúnez R., González-Gálvez N., Vale R.G.d.S., Martínez-Rodríguez A. (2019). Effects of a moderate-to-high intensity resistance circuit training on fat mass, functional capacity, muscular strength, and quality of life in elderly: A randomized controlled trial. Sci. Rep..

[18] Chiacchiero M., Dresely B., Silva U., DeLosReyes R., Vorik B. (2010). The relationship between range of movement, flexibility, and balance in the elderly. Top. Geriatr. Rehabil..

[19] Mueck-Weymann M., Janshoff G., Mueck H. (2004). Stretching increases heart rate variability in healthy athletes complaining about limited muscular flexibility. Clin. Auton. Res..

[20] Montero-Marín J., Asún S., Estrada-Marcén N., Romero R., Asún R. (2013). Effectiveness of a stretching program on anxiety levels of workers in a logistic platform: A randomized controlled study. Aten. Primaria.

[21] Hearing C.M., Chang W.C., Szuhany K.L., Deckersbach T., Nierenberg A.A., Sylvia L.G. (2016). Physical exercise for treatment of mood disorders: A critical. Curr. Behav. Neurosci. Rep..

[22] Baek S.-S. (2016). Role of exercise on the Brain. J. Exerc. Rehabil..

[23] Ruegsegger G.N., Booth F.W. (2018). Health benefits of exercise. Cold Spring Harb. Perspect. Med..

[24] Anderson E., Shivakumar G. (2013). Effects of exercise and physical activity on anxiety. Front. Psychiatry.

[25] Bassett-Gunter R., McEwan D., Kamarhie A., Anderson E., Shivakumar G. (2017). Physical activity and body image among men and boys: A meta-analysis. Body Image.

[26] Zelle D.M., Klaassen G., Van Adrichem E., Bakker S.J.L., Corpeleijn E., Navis G. (2017). Physical inactivity: A risk factor and target for intervention in renal care. Nat. Rev. Nephrol..

[27] Blair S.N. (2009). Physical inactivity: The biggest public health problem of the 21st Century. Br. J. Sports Med..

[28] World Health Organization (2019). Global Action Plan on Physical Activity 2018–2030: More Active People for a Healthier World.

[29] Guthold R., Ono T., Strong K.L., Chatterji S., Morabia A. (2008). Worldwide variability in physical inactivity. A 51-country survey. Am. J. Prev. Med..

[30] Ammar A., Brach M., Trabelsi K., Chtourou H., Boukhris O., Masmoudi L., Bouaziz B., Bentlage E., How D., Ahmed M. (2020). Effects of COVID-19 home confinement on eating behaviour and physical activity: Results of the ECLB-COVID 19 international online survey. Nutrients.

[31] Tison G.H., Avram R., Kuhar P., Abreau S., Marcus G.M., Pletcher M.J., Olgin J.E. (2020). Worldwide effect of COVID-19 on physical activity: A descriptive study. Ann. Intern. Med..

[32] Holtermann A., Straker L., Lee I.-M., Stamatakis E., Van Der Beek A.J. (2021). Workplace physical activity promotion: Why so many failures and few successes? The need for new thinking. Br. J. Sports Med..

[33] Bull F.C., Al-Ansari S.S., Biddle S., Borodulin K., Buman M.P., Cardon G., Carty C., Chaput J.P., Chastin S., Chou R. (2020). World health organization 2020 guidelines on physical activity and sedentary behaviour. Br. J. Sports Med..

[34] McEachan R.R.C., Lawton R.J., Jackson C., Conner M., Meads D.M., West R.M. (2011). Testing a workplace physical activity intervention: A cluster randomized controlled trial. Int. J. Behav. Nutr. Phys. Act..

[35] Grimani A., Aboagye E., Kwak L. (2019). The effectiveness of workplace nutrition and physical activity interventions in improving productivity, work performance and workability: A systematic review. BMC Public Health.

[36] Pronk N.P. (2021). Implementing movement at the workplace: Approaches to increase physical activity and reduce sedentary behavior in the context of work. Prog. Cardiovasc. Dis..

[37] Ntoumanis N., Thørgersen-Ntoumani C., Quested E., Chatzisarantis N. (2018). Theoretical approaches to physical activity promotion. Oxford Research Encyclopedia of Psychology.

[38] Bardus M., Blake H., Lloyd S., Suggs L.S. (2014). Reasons for participating and not participating in a E-health workplace physical activity intervention a qualitative analysis. Int. J. Work. Health Manag..

[39] Deci E.L., Ryan R.M. (2012). Self-determination theory. Handb. Theor. Soc. Psychol..

[40] Gagné M. (2014). The Oxford Handbook of Work Engagement and Self-Determination Theory.

[41] Deci E.L., Olafsen A.H., Ryan R.M. (2017). Self-determination theory in work organizations: The state of a science. Annu. Rev. Organ. Psychol. Organ. Behav..

[42] Teixeira P.J., Carraça E.V., Markland D., Silva M.N., Ryan R.M. (2012). Exercise, physical activity, and self-determination theory: A systematic review. Int. J. Behav. Nutr. Phys. Act..

[43] Tosi H.L., Locke E.A., Latham G.P. (1991). A theory of goal setting and task performance. Acad. Manag. Rev..

[44] Brehm J.W. (1989). The intensity of motivation. Ann. Rev. Psychol..

[45] Wright R.A. (2008). Refining the prediction of effort: Brehm’s distinction between potential motivation and motivation intensity. Soc. Personal. Psychol. Compass.

[46] Maria K.-M., George M. (2015). Workplace design: Conceptualizing and measuring workplace characteristics for motivation. J. Organ. Eff. People Perform..

[47] Sugiyama T., Hadgraft N.T., Healy G.N., Owen N., Dunstan D.W. (2019). Perceived availability of office shared spaces and workplace sitting: Moderation by organizational norms and behavioral autonomy. Environ. Behav..

[48] Le Deist F.D., Winterton J. (2005). What is competence?. Hum. Resour. Dev. Int..

[49] Butterworth S., Linden A., McClay W., Leo M.C. (2006). Effect of motivational interviewing-based health coaching on employees’ physical and mental health status. J. Occup. Health Psychol..

[50] Baicker K., Cutler D., Song Z. (2010). Workplace wellness programs can generate savings. Health Aff..

[51] Blackwell J., Collins M., Scribner C., Guillen J., Moses K., Gregory-Mercado K. (2019). Health and wellness coaching implemented by trainees: Impact in worksite wellness. Glob. Adv. Health Med..

[52] Carlsson C., Walden P. (2019). Digital Support to Guide Physical Activity-Augmented Daily Routines for Young Elderly. BLED 2019 Proceedings (Online). https://aisel.aisnet.org/bled2019/20/.

[53] Commissaris D.A., Huysmans M.A., Mathiassen S.E., Srinivasan D., Koppes L.L., Hendriksen I.J. (2016). Interventions to reduce sedentary behavior and increase physical activity during productive work: A systematic review. Scand. J. Work. Environ. Health.

[54] Maylor B.D., Edwardson C.L., Zakrzewski-Fruer J.K., Champion R.B., Bailey D.P. (2018). Efficacy of a multicomponent intervention to reduce workplace sitting time in office workers a cluster randomized controlled trial. J. Occup. Environ. Med..

[55] Nooijen C.F., Blom V., Ekblom Ö., Ekblom M.M., Kallings L.V. (2019). Improving office workers mental health and cognition a 3-Arm cluster randomized controlled trial targeting physical activity and sedentary behavior in multicomponent interventions. BMC Public Health.

[56] Rikli R.E., Jones C.J. (2013). Senior Fitness Test Manual.

[57] Manganelli L., Thibault-Landry A., Forest J., Carpentier J. (2018). Self-determination theory can help you generate performance and well-being in the workplace: A review of the literature. Adv. Dev. Hum. Resour..

[58] Invernizzi P.L., Signorini G., Bosio A., Scurati R. (2021). Children over “-Enty,-Rty,-Fty”: Gamification and autonomy as an environmental education leitmotif for “children of all ages” using a new workplace narrative. J. Phys. Educ. Sport.

[59] FitzGerald S.J., Barlow C.E., Kampert J.B., Morrow J.R., Jackson A.W., Blair S.N. (2004). Muscular fitness and all-cause mortality: Prospective observations. J. Phys. Act. Health.

[60] Micheo W., Baerga L., Miranda G. (2012). Basic principles regarding strength, flexibility, and stability exercises. Pm&r.

[61] Lee D.C., Artero E.G., Sui X., Blair S.N. (2010). Mortality trends in the general population: The importance of cardiorespiratory fitness. J. Psychopharmacol..

[62] Al-Mallah M.H., Sakr S., Al-Qunaibet A. (2018). Cardiorespiratory fitness and cardiovascular disease prevention: An update. Curr. Atheroscler. Rep..

[63] Lopez A.D., Mathers C.D., Ezzati M., Jamison D.T., Murray C.J. (2006). Global and regional burden of disease and risk factors, 2001: Systematic analysis of population health data. Lancet.

[64] World Health Organization (2002). The World Health Report 2002: Reducing Risks, Promoting Healthy Life.

[65] Katzmarzyk P.T., Craig C.L. (2002). Musculoskeletal fitness and risk of mortality. Med. Sci. Sports Exerc..

[66] Cunha AC V., Burke T.N., França FJ R., Marques A.P. (2008). Effect of global posture reeducation and of static stretching on pain, range of motion, and quality of life in women with chronic neck pain: A randomized clinical trial. Clinics.

[67] Papini G., Bonomi A.G., Stut W., Kraal J.J., Kemps H.M.C., Sartor F. (2017). Proof of concept of a 45-second cardiorespiratory fitness self-test for coronary artery disease patients based on accelerometry. PLoS ONE.

[68] Crotti M., Bosio A., Invernizzi P.L. (2018). Validity and reliability of submaximal fitness tests based on perceptual variables. J. Sports Med. Phys. Fitness.

[69] Invernizzi P.L., Signorini G., Bosio A., Raiola G., Scurati R. (2020). Validity and reliability of self-perception-based submaximal fitness tests in young adult females: An educational perspective. Sustainability.

[70] Harre D. (1997). Teoria Dell’allenamento.

[71] Freitas S.R., Vaz J.R., Gomes L., Silvestre R., Hilário E., Cordeiro N., Carnide F., Pezarat-Correia P., Mil-Homens P. (2015). A new tool to assess the perception of stretching intensity. J. Strength Cond. Res..

[72] Jones C.J., Rikli R.E., Max J., Noffal G. (1998). Cluarterly for exercise and sport a the reliability and validity of a chair sit-and-reach test as a measure of hamstring flexibility in older adults. Phys. Educ. Recreat. Danc..

[73] Craig C.L., Marshall A.L., Sjöström M., Bauman A.E., Booth M.L., Ainsworth B.E., Pratt M., Ekelund U., Yngve A., Sallis J.F. (2003). International physical activity questionnaire: 12-country reliability and validity. Med. Sci. Sports Exerc..

[74] Hart S.G., Staveland L.E. (1988). Development of NASA-TLX (Task Load Index): Results of empirical and theoretical research. Adv. Psychol..

[75] Hart S.G. (2006). NASA-task load index (NASA-TLX); 20 years later. Proc. Hum. Factors Ergon. Soc..

[76] Hoonakker P., Carayon P., Gurses A.P., Brown R., Khunlertkit A., McGuire K., Walker J.M. (2011). Measuring workload of ICU nurses with a questionnaire survey: The NASA task load index (TLX). IIE Trans. Healthc. Syst. Eng..

[77] Kodraliu G., Mosconi P., Groth N., Carmosino G., Perilli A., Gianicolo E.A., Rossi C., Apolone G. (2001). Subjective health status assessment: Evaluation of the italian version of the sf-12 health survey. Results from the MiOS project. J. Epidemiol. Biostat..

[78] Arvidsson D., Fridolfsson J., Buck C., Ekblom Ö., Ekblom-Bak E., Lissner L., Hunsberger M., Börjesson M. (2019). Reexamination of accelerometer calibration with energy expenditure as criterion: VO2net instead of MET for age-equivalent physical activity intensity. Sensors.

[79] Doherty A., Jackson D., Hammerla N., Plötz T., Olivier P., Granat M.H., White T., Van Hees V.T., Trenell M.I., Owen C.G. (2017). Large scale population assessment of physical activity using wrist worn accelerometers: The UK biobank study. PLoS ONE.

[80] Dieu O., Mikulovic J., Fardy P.S., Bui-Xuan G., Béghin L., Vanhelst J. (2017). Physical activity using wrist-worn accelerometers: Comparison of dominant and non-dominant Wrist. Clin. Physiol. Funct. Imaging.

[81] Kenttä G., Hassmén P. (1998). Overtraining and recovery. Sport. Med..

[82] Foster C., Daines E., Hector L., Snyder A.C., Welsh R. (1996). Athletic performance in relation to training load. Wis. Med. J..

[83] Bakeman R. (2005). Recommended effect size statistics for repeated measures designs. Behav. Res. Methods.

[84] Cohen J. (2013). Statistical Power Analysis for the Behavioral Sciences.

[85] Santos E., Rhea M., Simão R., Dias I., De Salles B.F., Novaes J., Leite T., Blair J.C., Bunker D.J. (2010). Influence of moderately intense strength training on flexibility in sedentary young women. J. Strength Cond. Res..

[86] Childs J.D., Teyhen D.S., Benedict T.M., Morris J.B., Fortenberry A.D., McQueen R.M., Preston J.B., Wright A.C., Dugan J.L., George S.Z. (2009). Effects of Sit-up training versus core stabilization exercises on sit-up performance. Med. Sci. Sports Exerc..

[87] Baxter R.E., Moore J.H., Pendergrass T.L., Crowder T.A., Lynch S. (2003). Improvement in sit-up performance associated with 2 different training regimens. J. Orthop. Sport. Phys. Ther..

[88] Shariat A., Ghannadi S., Anastasio A.T., Rostad M., Cleland J.A. (2020). Novel stretching and strength-building exercise recommendations for computer-based workers during the COVID-19 quarantine. Work.

[89] Boulé N.G., Kenny G.P., Haddad E., Wells G.A., Sigal R.J. (2003). Meta-analysis of the effect of structured exercise training on cardiorespiratory fitness in type 2 diabetes mellitus. Diabetologia.

[90] Branch J.D., Pate R.R., Bourque S.P. (2000). Moderate intensity exercise training improves cardiorespiratory fitness in women. J. Women’s Health Gender-Based Med..

[91] Dunn A.L., Marcus B.H., Kampert J.B., Garcia M.E., Kohl H.W., Blair S.N. (1999). Comparison of lifestyle and structured interventions to increase physical activity and cardiorespiratory fitness: A randomized trial. J. Am. Med. Assoc..

[92] Chen H., Chung Y., Chen Y., Ho S., Wu H. (2017). Effects of different types of exercise on body composition, muscle strength, and IGF-1 in the elderly with sarcopenic obesity. J. Am. Geriatr. Soc..

[93] Kopp M.S., Stauder A., Purebl G., Janszky I., Skrabski Á. (2008). Work stress and mental health in a changing society. Eur. J. Public Health.

[94] Taelman J., Vandeput S., Spaepen A., Van Huffel S. (2008). Influence of mental stress on heart rate and heart rate variability. IFMBE Proc..

[95] Wahlström J., Lindegård A., Ahlborg G., Ekman A., Hagberg M. (2003). Perceived muscular tension, emotional stress, psychological demands and physical load during VDU work. Int. Arch. Occup. Environ. Health.

[96] Wandel M., Roos G. (2005). Work, food and physical activity. A qualitative study of coping strategies among men in three occupations. Appetite.

[97] Faulkner G., Rhodes R.E., Vanderloo L.M., Chulak-Bozer T., O’Reilly N., Ferguson L., Spence J.C. (2020). Physical activity as a coping strategy for mental health due to the COVID-19 virus: A potential disconnect among canadian adults?. Front. Commun..

[98] Dyrstad S.M., Hansen B.H., Holme I.M., Anderssen S.A. (2013). Comparison of self-reported versus accelerometer-measured physical activity. Med. Sci. Sports Exerc..

[99] Marcora S.M., Bruce G.E. (2010). Effort: Perception of. Encyclopedia of Perception.

[100] Lindberg C.M., Srinivasan K., Gilligan B., Razjouyan J., Lee H., Najafi B., Mehl M.R., Currim F., Ram S., Lunden M.M. (2018). Effects of office workstation type on physical activity and stress. Occup. Environ. Med..

[101] Beauchemin J., Lee M.Y. (2014). Solution-focused. Wellness coaching. J. Solut. Focus. Pract..

